# Differentiating Neoplastic From Non-neoplastic Gallbladder Lesions Using MUC1 and MUC5AC: An Immunohistochemical Analysis

**DOI:** 10.7759/cureus.88040

**Published:** 2025-07-15

**Authors:** Umika Gupta, Vijai Singh, Sanjeev Yadav

**Affiliations:** 1 Department of Pathology, Hind Institute of Medical Sciences, Sitapur, IND

**Keywords:** biomarkers, cholecystitis, gallbladder neoplasms, immunohistochemistry, muc1, muc5ac

## Abstract

Background: Gallbladder lesions range from benign inflammatory conditions to malignancies, often progressing through pre-neoplastic stages. Accurate histopathological differentiation, particularly in early stages, remains challenging. Mucin proteins MUC1 and MUC5AC have emerged as potential immunohistochemical (IHC) markers for distinguishing neoplastic from non-neoplastic gallbladder pathology.

Material and methods: A cross-sectional study was conducted on 52 formalin-fixed, paraffin-embedded (FFPE) gallbladder specimens from cholecystectomy cases over two years. Routine histopathological evaluation was followed by IHC staining using monoclonal antibodies against MUC1 and MUC5AC. Expression levels were semi-quantitatively scored (0-3) and statistically analyzed in relation to neoplastic status.

Results: Of the 52 cases, 29 (55.8%) were non-neoplastic and 23 (44.2%) were neoplastic, with 91.3% (n = 21) of the latter being malignant. MUC1 expression was significantly higher in neoplastic lesions (p < 0.001), with 73.9% (n = 17) showing strong (score 3) positivity. Conversely, MUC5AC was predominantly expressed in non-neoplastic lesions, with strong expression observed in 55.2% (n = 16) versus 13.0% (n = 3) of neoplastic cases (p < 0.001). MUC1 demonstrated high diagnostic performance for neoplasia (sensitivity 95.7%, specificity 75.9%), while MUC5AC showed moderate performance (sensitivity 69.6%, specificity 72.4%). The inverse expression profiles of these markers effectively differentiated malignant from benign lesions.

Conclusion: MUC1 and MUC5AC exhibit distinct IHC expression patterns in gallbladder pathology. MUC1 is a sensitive marker for neoplastic transformation, whereas MUC5AC is more indicative of benign or early-stage lesions. Their combined application may improve diagnostic accuracy in histologically ambiguous cases. Incorporating these biomarkers into routine pathology practice may aid early detection and better clinical decision-making. Further large-scale studies are needed to validate their prognostic significance.

## Introduction

Gallbladder disease (GBD) represents a substantial public health concern worldwide, contributing significantly to abdominal morbidity and mortality. It has been reported as an emerging burden, particularly in developed countries, where lifestyle-related risk factors have contributed to a rising incidence [1]. GBD includes a diverse range of pathological conditions, encompassing inflammatory (e.g., acute and chronic cholecystitis), metabolic (e.g., cholesterolosis), and neoplastic processes (e.g., adenomas, adenocarcinomas) [2]. Histopathological evaluation often reveals a spectrum of mucosal alterations, ranging from simple inflammation to pre-neoplastic and malignant transformations. Epidemiologically, GBD affects both sexes but shows higher prevalence in women, particularly during pregnancy and in individuals with obesity, advanced age (especially in their 40s and 50s), rapid weight loss, or metabolic syndrome [3].

The pathogenesis of GBD is primarily initiated by obstruction of the cystic duct or dysregulated gallbladder motility, leading to bile stasis, mucosal injury, and secondary inflammation [4]. This initiates a cascade of pathological changes such as venous congestion, edema, infiltration of inflammatory cells, and ultimately fibrosis and chronic inflammation. In severe cases, this may progress to complications such as gallbladder gangrene, perforation, or sepsis. Histologically, gallbladder lesions are commonly classified into three broad categories: inflammatory, including chronic and acute cholecystitis and their variants; pre-neoplastic, such as pyloric or intestinal metaplasia and adenomatous hyperplasia; and neoplastic, including benign tumors and carcinomas such as adenocarcinoma and papillary carcinoma [5,6]. This continuum from inflammation to neoplasia underscores the need for early identification and monitoring of precursor lesions to prevent malignant progression.

One of the major challenges in gallbladder pathology is the early and accurate diagnosis of pre-neoplastic and early neoplastic changes. Unlike well-characterized precursor lesions in organs such as the cervix, colon, or pancreas, the precursor lesions of gallbladder carcinoma remain poorly understood. The clinical and pathological features of these lesions are under-characterized, partly due to their rarity, inconsistent diagnostic terminology, and the absence of standardized classification systems [4]. This diagnostic ambiguity can delay appropriate therapeutic intervention and contribute to the poor prognosis associated with gallbladder cancer, which is often detected at an advanced stage. Consequently, there is a critical need for robust biomarkers that can facilitate the detection and stratification of these lesions based on their malignant potential.

Mucins are high-molecular-weight glycoproteins produced by epithelial tissues and serve key roles in cell signaling, adhesion, and protection of epithelial surfaces. They are broadly classified into membrane-bound mucins (e.g., MUC1, MUC4) and secretory or gel-forming mucins (e.g., MUC2, MUC5AC). In the context of gallbladder pathology, mucins have gained attention as potential diagnostic and prognostic markers. MUC1 is a transmembrane mucin that is typically overexpressed in invasive carcinomas and is associated with epithelial-to-mesenchymal transition, tumor invasion, and poor prognosis [7,8]. It has been linked with lymph node metastasis, loss of cellular polarity, and aggressive tumor behavior. MUC5AC, on the other hand, is a secretory mucin normally expressed in the gastric epithelium and is often upregulated in gallbladder inflammatory and pre-neoplastic lesions. Its expression tends to diminish with increasing dysplasia and carcinoma, suggesting its role in early mucosal alterations rather than in malignancy [9].

Several studies have evaluated the expression of MUC1 and MUC5AC in gallbladder tissues and found significant associations with histological subtype and clinical outcomes. However, current literature is limited by small sample sizes, heterogeneity in case selection, and lack of correlation with comprehensive histological classifications [7,10,11]. These limitations necessitate further investigations that not only examine MUC1 and MUC5AC expression patterns across a wider histopathological spectrum but also correlate them with disease severity and progression.

The present study aims to fill this gap by evaluating the distribution of gallbladder lesions in a tertiary care setting and by analyzing the immunohistochemical (IHC) expression patterns of MUC1 and MUC5AC. Specifically, the study seeks to correlate these expression profiles with the histopathological classification of GBDs, ranging from non-neoplastic to malignant lesions. By identifying distinct expression patterns, this research aims to enhance diagnostic accuracy and potentially contribute to improved clinical management and prognostication in gallbladder disease.

## Materials and methods

Study design and participants

This cross-sectional observational study was conducted over two years (2020-2022) in the Department of Pathology, Hind Institute of Medical Sciences, Sitapur, India. Ethical clearance was obtained from the Institutional Ethics Committee, and informed written consent was obtained from all participants. The study included 52 formalin-fixed, paraffin-embedded (FFPE) gallbladder tissue specimens obtained from patients who underwent elective cholecystectomy. The majority of the procedures were laparoscopic cholecystectomies (n = 25, 48.1%), in line with standard surgical practice for benign GBD, while open cholecystectomy was performed in 27 (51.9%) cases. These cases were submitted for routine histopathological evaluation. The specimens included were those obtained from patients undergoing cholecystectomy for GBD, while inadequate or autolyzed samples and those lacking clinical information were excluded. 

Tissue processing and histopathological evaluation

Demographic and clinical details were recorded for each case. The gallbladder specimens were grossly examined to assess the mucosa, wall thickness, and serosal surface. Representative tissue sections were obtained from key anatomical regions, including the fundus, body, and neck. In cases with suspected tumors, additional sections were taken from the cystic duct margin and nearby lymph nodes for comprehensive evaluation. The tissues were routinely processed: fixed in formalin, embedded in paraffin, sectioned at 4-5 µm, and stained with hematoxylin and eosin (H&E). These procedures were carried out according to standard histopathological laboratory protocols, as generally recommended in diagnostic pathology references [12,13].

Immunohistochemistry (IHC)

IHC staining was performed to assess the expression of MUC1 and MUC5AC mucin proteins. Sections were cut at 4-5 µm, mounted on poly-L-lysine-coated slides, and dried at 60°C. Deparaffinization and rehydration were carried out using standard xylene and graded ethanol series. Antigen retrieval was performed in citrate buffer (pH 6.0) using a pressure cooker. Endogenous peroxidase activity was quenched using 3% hydrogen peroxide.

Primary antibodies used were mouse monoclonal antibodies for MUC1 (Clone BSB-44, Bio-SB, USA) and MUC5AC (Clone CLH-2, Bio-SB, USA). Incubation and dilutions followed the manufacturer's recommendations. Signal detection was performed using a horseradish peroxidase (HRP)-linked secondary antibody system with 3,3′-diaminobenzidine (DAB) as chromogen. Sections were counterstained with hematoxylin and mounted using DPX. Positive controls (kidney for MUC1 and stomach for MUC5AC) and negative controls (omission of primary antibody) were included. MUC1 staining was localized to the luminal membrane and cytoplasm, while MUC5AC expression was predominantly cytoplasmic [7]. Only cytoplasmic staining was considered for scoring.

Scoring of immunostaining

IHC expression of MUC1 and MUC5AC was evaluated using a semi-quantitative approach that considered both the percentage of positively stained epithelial cells and the intensity of staining. A composite score ranging from 0 to 3 was assigned: 0 denoted no staining or less than 10% of cells stained; 1 represented weak staining in 10%-25% of cells; 2 indicated moderate staining in 26%-50% of cells; and 3 corresponded to strong staining in more than 50% of cells. Only cytoplasmic or membranous staining, depending on the marker’s biological localization, was included in the evaluation. Two independent pathologists, blinded to clinical and histopathological information, reviewed all slides to reduce observer bias. This scoring method reflects commonly accepted practices for mucin IHC evaluation in gallbladder cancer research [7].

Statistical analysis

Data were analyzed using SPSS Statistics for Windows, Version 15.0 (Released 2006; SPSS Inc., Chicago, IL, US). Descriptive statistics were used for demographic variables. Chi-square test or Fisher’s exact test was applied to categorical variables. Diagnostic performance was assessed using 2 × 2 contingency tables, calculating sensitivity, specificity, positive predictive value (PPV), negative predictive value (NPV), and diagnostic accuracy. A p-value of <0.05 was considered statistically significant.

## Results

The present study included 52 cases with a mean age of 47.23 ± 10.71 years (range: 25-74 years). The majority of patients belong to the 31-40 years age group (n = 16, 30.8%), followed by 51-60 years (n = 15, 28.8%) and 41-50 years (26.9%) (Table 1). Only 3.8% (n = 2) of patients were aged ≤30 years, while 9.6% (n = 5) were above 60. There was a marked female predominance, with females constituting 76.9% (n = 40) of the study population and males 23.1% (n = 12). Of the total cases, 29 (55.8%) were categorized as non-neoplastic, while 23 cases (44.2%) were neoplastic. Among the non-neoplastic lesions, chronic cholecystitis was the most common histopathological diagnosis, observed in nine cases (31.0%). Other non-neoplastic entities included chronic cholecystitis with cholesterolosis (n = 4, 13.8%), chronic cholecystitis with intestinal metaplasia (n = 4, 13.8%), chronic cholecystitis with pyloric metaplasia (n = 4, 13.8%), follicular cholecystitis (n = 2, 6.9%), xanthogranulomatous cholecystitis (n = 2, 6.9%), hyalinizing cholecystitis (n = 1, 3.4%), acute cholecystitis (n = 1, 3.4%), acute chronic cholecystitis (n = 1, 3.4%), and mucocele (n = 1, 3.4%). Among the neoplastic lesions, malignant tumors constituted the vast majority, accounting for 91.3% (21/23), while only one benign lesion (4.4%) and one case of adenomatous hyperplasia (4.4%) were identified. The malignant cases predominantly comprised adenocarcinomas (n = 18, 85.7%), followed by papillary adenocarcinoma (n = 2, 9.5%) and a single case of intracholecystic papillary neoplasm (ICPN) with associated invasive carcinoma (n = 1, 4.8%). In carcinoma cases, lymph node metastasis was observed in 6 out of 15 cases (40.0%). Lymphovascular invasion (LVI) and perineural invasion (PNI) were each noted in 3 out of 20 cases (15.0%). Tumor-infiltrating lymphocytes (TILs) showed mild infiltration in eight cases (40.0%), moderate in seven cases (35.0%), marked in three cases (15.0%), and were absent in two cases (10.0%). Tumor necrosis was present in 9 out of 20 carcinoma cases (45.0%) (Table 1).

**Table 1:** Demographic, histopathological, and clinicopathological characteristics of the study population

Characteristic	n (%)
Mean age ± SD (range)	47.23 ± 10.71 (25-74)
Age group	
≤30 years	2 (3.8%)
31-40 years	16 (30.8%)
41-50 years	14 (26.9%)
51-60 years	15 (28.8%)
>60 years	5 (9.6%)
Sex	
Male	12 (23.1%)
Female	40 (76.9%)
Neoplasm status	
Non-neoplastic	29 (55.8%)
Neoplastic	23 (44.2%)
Non-neoplastic lesions (n = 29)	
Chronic cholecystitis	9 (31.0%)
Chronic cholecystitis with cholesterolosis	4 (13.8%)
Chronic cholecystitis with intestinal metaplasia	4 (13.8%)
Chronic cholecystitis with pyloric metaplasia	4 (13.8%)
Follicular cholecystitis	2 (6.9%)
Xanthogranulomatous cholecystitis	2 (6.9%)
Hyalinizing cholecystitis	1 (3.4%)
Acute cholecystitis	1 (3.4%)
Acute on chronic cholecystitis	1 (3.4%)
Mucocele	1 (3.4%)
Neoplastic lesions (n = 23)	
Benign	1 (4.4%)
Adenomatous hyperplasia	1 (4.4%)
Malignant	21 (91.3%)
Malignant (n = 21)	
Adenocarcinoma	18 (85.7%)
Papillary adenocarcinoma	2 (9.5%)
Intracholecystic papillary neoplasm (ICPN)	1 (4.8%)
Pathological features in carcinoma cases	
Lymph node metastasis (n = 15)	6 (40.0%)
Lymphovascular invasion (LVI) (n = 20)	3 (15.0%)
Perineural invasion (PNI) (n = 20)	3 (15.0%)
Tumor-infiltrating lymphocytes (TILs) (n = 20)	
Negative	2 (10.0%)
Mild	8 (40.0%)
Moderate	7 (35.0%)
Marked	3 (15.0%)
Tumor necrosis (n = 20)	9 (45.0%)

Distribution and comparative analysis of MUC1 and MUC5AC expressions

**Figure 1:**
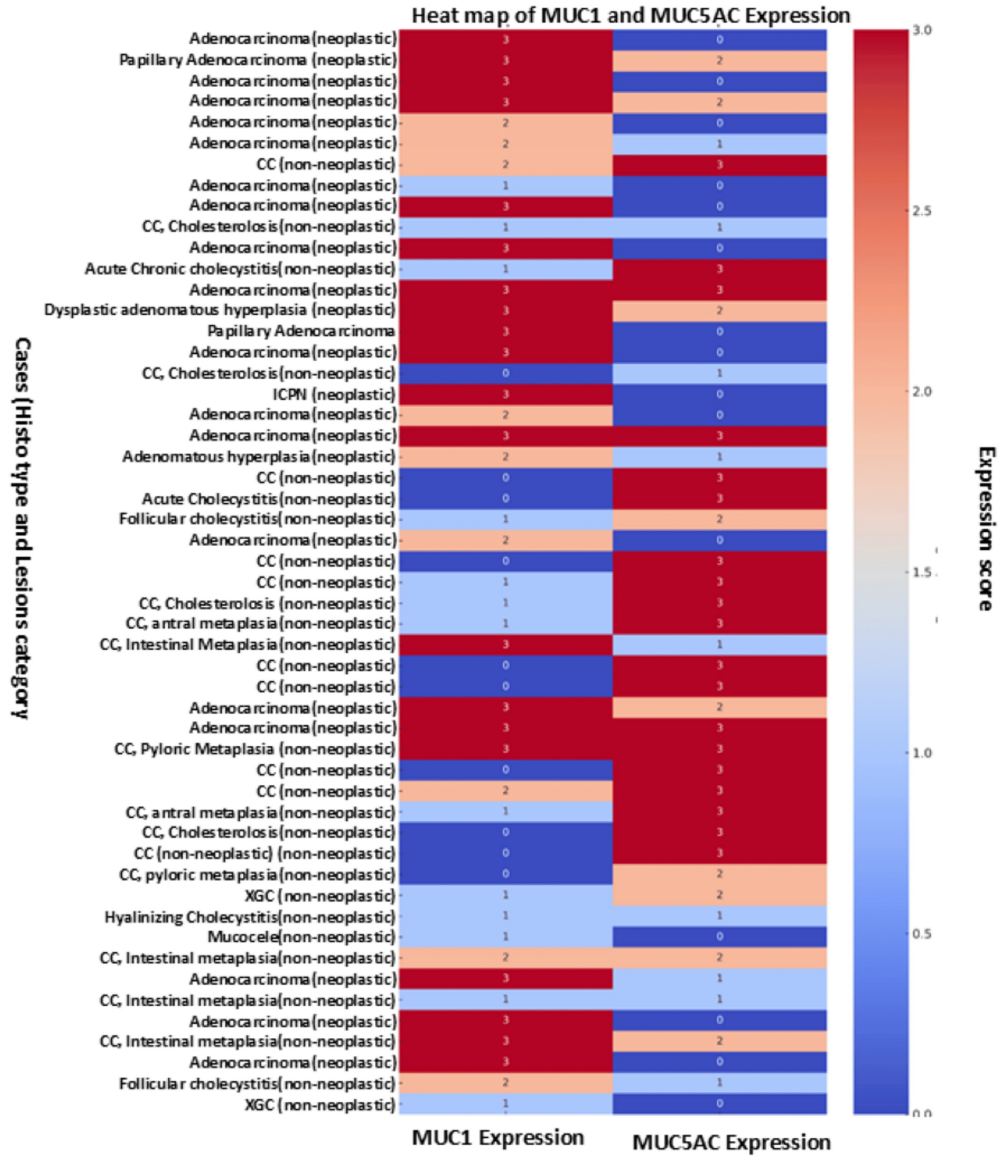
Heatmap showing MUC1 and MUC5AC expressions across all histological cases Each row represents a case labelled by its histology type and lesion category (neoplastic or non-neoplastic). Colors reflect expression intensity: red shades indicate higher expression and blue shades indicate lower expression.

IHC expression of MUC1 was observed in 42 out of 52 cases (80.8%), while MUC5AC expression was identified in 37 cases (71.2%). Among MUC1-positive specimens, the majority showed a strong expression (score 3; n = 20, 38.5%), followed by weak expression (score 1; n = 13, 25.0%) and moderate expression (score 2; n = 9, 17.3%). Similarly, for MUC5AC-positive cases, the highest proportion exhibited strong expression (score 3; n = 19, 36.5%), whereas nine cases each (17.3%) demonstrated weak (score 1) and moderate (score 2) staining intensity (Table 2; Figure 1).

**Table 2:** Immunohistochemical expression of MUC1 and MUC5AC in gallbladder lesions

Marker	IHC score	Total cases (n = 52)	Non-neoplastic (n = 29)	Neoplastic (n = 23)	p-value (neoplastic vs. non-neoplastic)
MUC1	0	10 (19.2%)	10 (34.5%)	0 (0.0%)	<0.001
	1	13 (25.0%)	12 (41.4%)	1 (4.3%)	
	2	9 (17.3%)	4 (13.8%)	5 (21.7%)	
	3	20 (38.5%)	3 (10.3%)	17 (73.9%)	
MUC5AC	0	15 (28.8%)	2 (6.9%)	13 (56.5%)	<0.001
	1	9 (17.3%)	6 (20.7%)	3 (13.0%)	
	2	9 (17.3%)	5 (17.2%)	4 (17.4%)	
	3	19 (36.5%)	16 (55.2%)	3 (13.0%)	

When comparing MUC1 expression between non-neoplastic and neoplastic gallbladder lesions, a significant difference was noted. In the non-neoplastic group (n = 29), the majority of cases exhibited either no expression (score 0; n = 10, 34.5%) or weak expression (score 1; n = 12, 41.4%), with only a small number showing moderate (score 2; n = 4, 13.8%) or strong expression (score 3; n = 3, 10.3%). Conversely, in the neoplastic group (n = 23), none of the cases had a score of 0, and the vast majority exhibited strong expression (score 3; n = 17, 73.9%). Only a few cases demonstrated weak (score 1; n = 1, 4.3%) or moderate expression (score 2; n = 5, 21.7%). This difference in MUC1 expression between neoplastic and non-neoplastic groups was statistically significant (Mann-Whitney U test: z = 5.199; p < 0.001).

Similarly, MUC5AC expression patterns also differed significantly between the two groups. Among non-neoplastic lesions, a predominant number of cases demonstrated strong expression (score 3; n = 16, 55.2%), followed by weak (score 1; n = 6, 20.7%), moderate (score 2; n = 5, 17.2%), and absent expression (score 0; n = 2, 6.9%). In contrast, neoplastic cases showed a reverse trend, with a majority showing no expression (score 0; n = 13, 56.5%). Fewer cases displayed weak (score 1; n = 3, 13.0%), moderate (score 2; n = 4, 17.4%), or strong expression (score 3; n = 3, 13.0%). This variation in MUC5AC expression between non-neoplastic and neoplastic lesions was also statistically significant (Mann-Whitney U test: z = 3.857; p < 0.001) (Table 2).

Diagnostic performance of MUC1 and MUC5AC IHC expression in differentiating neoplastic and non-neoplastic gallbladder lesions (n = 52)

The diagnostic efficacy of MUC1 and MUC5AC expression was evaluated to differentiate between neoplastic and non-neoplastic gallbladder lesions. For MUC1, an IHC score of 2 or 3 was considered positive for neoplastic lesions. Based on this cutoff, 22 (95.7%) cases were true positives, 7 (24.1%) were false positives, 1 (4.3%) was a false negative, and 22 (75.9%) were true negatives. This yielded a sensitivity of 95.7%, specificity of 75.9%, PPV of 75.9%, NPV of 95.7%, and an overall diagnostic accuracy of 84.6% (Table 3).

In contrast, for MUC5AC, an IHC score of 0 or 1 was considered indicative of neoplastic lesions. Using this threshold, 16 (69.6%) cases were true positives, 8 (27.6%) were false positives, 7 (30.4%) were false negatives, and 21 (72.4%) were true negatives. The corresponding diagnostic indices were sensitivity (69.6%), specificity (72.4%), PPV (66.7%), NPV (75.0%), and overall accuracy (71.2%). MUC1 has superior diagnostic performance compared to MUC5AC in distinguishing neoplastic from non-neoplastic gallbladder lesions, highlighting its potential utility as a reliable IHC biomarker in routine diagnostic practice (Table 3).

**Table 3:** Diagnostic performance of MUC1 and MUC5AC immunohistochemical expression in differentiating neoplastic and non-neoplastic gallbladder lesions

IHC marker	IHC score group	Neoplastic (n = 23)	Non-neoplastic (n = 29)	Total (n = 52)	Sensitivity (%)	Specificity (%)	PPV (%)	NPV (%)	Accuracy (%)
MUC1	Scores 2/3	22 (95.7%)	7 (24.1%)	29 (55.8%)	95.7	75.9	75.9	95.7	84.6
	Scores 0/1	1 (4.3%)	22 (75.9%)	23 (44.2%)					
MUC5AC	Scores 0/1	16 (69.6%)	8 (27.6%)	24 (46.2%)	69.6	72.4	66.7	75.0	71.2
	Scores 2/3	7 (30.4%)	21 (72.4%)	28 (53.8%)					

Comparison of MUC1 and MUC5AC IHC expression between malignant and non-malignant gallbladder lesions

A comparative analysis expression of MUC1 and MUC5AC was performed between malignant (n = 21) and non-malignant (n = 31) gallbladder lesions. The majority of malignant cases (n = 20/21; 95.2%) demonstrated high MUC1 expression, with IHC scores of 2 or 3. In contrast, the majority of non-malignant lesions (n = 22/31; 71.0%) exhibited low MUC1 expression, with IHC scores of 0 or 1. This difference in MUC1 expression between the two groups was statistically significant (Mann-Whitney U test: z = 4.921; p < 0.001) (Table 4). Regarding MUC5AC, most malignant lesions (n = 15/21; 71.4%) exhibited low expression (IHC scores 0 or 1), whereas the majority of non-malignant lesions (n = 22/31; 71.0%) showed higher expression levels (IHC scores 2 or 3). The difference in MUC5AC expression between malignant and non-malignant lesions was also statistically significant (Mann-Whitney U test: z = 3.826; p < 0.001) (Table 4).

**Table 4:** Distribution and comparative expression of MUC1 and MUC5AC among gallbladder lesions

IHC marker	IHC score	Total (n = 52)	Malignant (n = 21)	Non-malignant (n = 31)	p-value (malignant vs. non-malignant)
MUC1	0	10 (19.2%)	0 (0.0%)	10 (32.3%)	<0.001
	1	13 (25.0%)	1 (4.8%)	12 (38.7%)	
	2	9 (17.3%)	4 (19.0%)	5 (16.1%)	
	3	20 (38.5%)	16 (76.2%)	4 (12.9%)	
MUC5AC	0	15 (28.8%)	13 (61.9%)	2 (6.5%)	<0.001
	1	9 (17.3%)	2 (9.5%)	7 (22.6%)	
	2	9 (17.3%)	3 (14.3%)	6 (19.4%)	
	3	19 (36.5%)	3 (14.3%)	16 (51.6%)	

Evaluation of the diagnostic efficacy of MUC1 and MUC5AC for differentiating malignant from non-malignant gallbladder lesions

The diagnostic performance of MUC1 IHC expression was evaluated by considering scores 2 and 3 as the threshold for positivity. Based on this cut-off, 20 (95.2%) cases were true positives, 9 (29.0%) were false positives, 1 (4.8%) was false negative, and 22 (71.0%) were true negatives. The sensitivity, specificity, PPV, NPV, and diagnostic accuracy for MUC1 IHC in identifying malignant gallbladder lesions were 95.2%, 71.0%, 69.0%, 95.7%, and 80.8%, respectively (Table 5). Similarly, for MUC5AC IHC expression, scores 0 and 1 were considered indicative of malignancy. With this criterion, there were 15 (71.4%) true positives, 9 (29.0%) false positives, 6 (28.6%) false negatives, and 22 (71.0%) true negatives. The corresponding sensitivity, specificity, PPV, NPV, and accuracy of MUC5AC IHC were 75.0%, 71.0%, 62.5%, 78.6%, and 71.2%, respectively (Table 5).

**Table 5:** Evaluation of diagnostic efficacy of MUC1 and MUC5AC IHC expressions in differentiating malignant from non-malignant gallbladder lesions

IHC marker	IHC score group	Malignant (n = 21)	Non-malignant (n = 31)	Total (n = 52)	Sensitivity (%)	Specificity (%)	PPV (%)	NPV (%)	Accuracy (%)
MUC1	Scores 2/3	20 (95.2%)	9 (29.0%)	29 (55.8%)	95.2	71.0	69.0	95.7	80.8
	Scores 0/1	1 (4.8%)	22 (71.0%)	23 (44.2%)					
MUC5AC	Scores 0/1	15 (71.4%)	9 (29.0%)	24 (46.2%)	75.0	71.0	62.5	78.6	71.2
	Scores 2/3	6 (28.6%)	22 (71.0%)	28 (53.8%)					

## Discussion

The advent of IHC techniques has significantly enhanced the ability to investigate the genetic and molecular underpinnings of GBDs. These methods have proven particularly valuable in improving diagnostic accuracy, especially when conventional histomorphological assessment yields inconclusive results. Mucins are glycoproteins that play a pivotal role in epithelial cell protection, tissue homeostasis, and intracellular signaling. They create a microenvironment conducive to cancer progression by protecting cells from hypoxia, acidity, and other hostile conditions. Mucins are broadly categorized into secreted and transmembrane types, both of which are implicated in inflammation and carcinogenesis [14]. Their prognostic utility and potential as therapeutic targets have been recognized in various malignancies, although data specific to gallbladder carcinomas (GBCs) remain limited [14,15]. Although gallbladder lesions are predominantly non-neoplastic, chronic inflammation such as chronic cholecystitis may progress through a sequence of hyperplasia, metaplasia, and dysplasia, eventually culminating in malignancy [10,16]. This premalignant potential underscores the importance of studying mucin expression in routine cholecystectomy specimens.

In our study, patient ages ranged from 25 to 74 years, with a mean age of 47.23 ± 10.71 years, and a marked female predominance (76.9%). These demographic patterns align with findings from a study conducted in Varanasi, which reported that GBCs predominantly affect females in their fourth and fifth decades of life, with a male-to-female ratio of 1:3.37 [17]. Additionally, a prospective study at a tertiary care center observed a median age of 60 years among GBC patients, with 75% of patients having gallstones and 21% presenting with a history of chronic cholecystitis [18]. In our cohort, chronic cholecystitis emerged as the most common non-neoplastic lesion (n = 9, 17.3%), followed by chronic cholecystitis associated with cholesterolosis, intestinal metaplasia, and pyloric metaplasia (n = 4, 13.8% each). Other histologies included follicular cholecystitis (n = 2, 6.9%) and xanthogranulomatous cholecystitis (n = 2, 6.9%). These findings align with those of Pathak et al., who reported chronic cholecystitis in 68.5% of cases, and are supported by similar high frequencies in other studies [19-21].

Histopathologically, 55.8% (n = 29) of cases were non-neoplastic, predominantly chronic cholecystitis (n = 21), and 44.2% (n = 23) were neoplastic. Among the neoplastic cases, malignant lesions predominated (n = 21, 91.3%), with adenocarcinoma being the most common subtype (n = 18, 85.7%). Lymph node metastasis was noted in 40% (n = 6/15) of GBCs evaluated. Lymphovascular and perineural invasions were each observed in 15% (n = 3/20) of cases. Necrosis was documented in 45% (n = 9/20) of cases. Compared to our findings, Kashiwagi et al. reported lymphatic and venous invasion in 27.8% and 24.1% of GBC cases, respectively, and lymph node involvement in 18.5% cases [22]. Similarly, Bhoge et al. and Lee et al. reported lymph node metastases in 37.8% and 11.1% of GBC cases, respectively, highlighting the variability in the pathological spectrum across studies [7,23].

In our study, MUC1 expression showed a significantly higher prevalence and intensity in neoplastic lesions compared to non-neoplastic ones (p < 0.001). Notably, 73.9% (n = 17/23) of neoplastic lesions exhibited a strong MUC1 IHC score of 3, whereas only 10.3% (n = 3/29) of non-neoplastic lesions reached this expression level. Conversely, low or absent MUC1 expression (scores 0 and 1) was predominantly observed in non-neoplastic lesions (n = 22/29, 75.9%) and was rare in neoplastic cases (n = 1/23, 4.3%). These findings are consistent with previous reports suggesting that MUC1 overexpression is closely associated with malignant transformation in gallbladder tissues. Ghosh et al. found significantly higher MUC1 expression in both mucosal and submucosal layers of GBC cases compared to chronic cholecystitis and normal gallbladders [24]. Similarly, Maurya et al. reported stronger MUC1 immunoreactivity in advanced stages (T2 and above) of GBC, while non-neoplastic lesions and early-stage cancers (T1) showed weaker or absent expression [25]. Bhoge et al. reported MUC1 positivity in 85.7% of GBC cases, compared to just 4.7% of chronic cholecystitis cases, supporting its utility in distinguishing malignant from inflammatory lesions [7]. Collectively, these studies, including the present one, reinforce the role of MUC1 as a potential marker of malignancy in gallbladder pathology.

In contrast to MUC1, MUC5AC showed a reverse trend, with significantly higher expression in non-neoplastic lesions (p < 0.001). Over half of non-neoplastic cases (n = 16/29, 55.2%) demonstrated strong MUC5AC expression (score 3), while only 13% (n = 3) of neoplastic lesions exhibited a similar level of expression. Notably, a large proportion of neoplastic lesions (n = 13/23, 56.5%) showed complete absence of MUC5AC expression (score 0), compared to only 6.9% (n = 2/29) of non-neoplastic cases. This inverse relationship between MUC5AC expression and malignancy is supported by prior research. Xiong et al. observed MUC5AC expression in 94.3% of non-neoplastic and 80% of benign neoplastic lesions, but only 51.9% of malignant cases [10]. In the same vein, Bhoge et al. reported MUC5AC positivity in 87.2% of chronic cholecystitis cases but only 28.6% of GBC cases [7]. These observations suggest that MUC5AC expression is more closely aligned with inflammatory or benign conditions and tends to diminish with malignant transformation, possibly due to mucosal dedifferentiation or altered glycosylation pathways during carcinogenesis. The distinct immunohistochemical profiles of MUC1 and MUC5AC observed in this study underscore their complementary roles in differentiating neoplastic from non-neoplastic gallbladder lesions. While high MUC1 expression favors a malignant diagnosis, strong MUC5AC expression appears more indicative of non-neoplastic or inflammatory conditions. These markers could serve as valuable adjuncts in histopathological evaluation, particularly in equivocal cases or where malignancy is suspected but not morphologically evident.

The immunohistochemical scoring of MUC1 and MUC5AC was further analyzed to determine their diagnostic performance in distinguishing neoplastic from non-neoplastic gallbladder lesions (Table 3). This evaluation revealed that MUC1, when expressed at moderate to strong levels (scores 2/3), demonstrates a high sensitivity of 95.7% and a specificity of 75.9% for the identification of neoplastic lesions. In contrast, MUC5AC, when expressed at low levels (scores 0/1), yielded a more modest sensitivity of 69.6% and specificity of 72.4%.

These results are consistent with prior studies demonstrating the superior diagnostic accuracy of MUC1 over MUC5AC in differentiating malignant from benign gallbladder pathology. In the study by Bhoge et al., MUC1 positivity distinguished neoplastic from chronic calculous cholecystitis with 83.3% sensitivity and 93.5% specificity, and from acalculous cholecystitis with the same sensitivity and 100% specificity [7]. Although specificity in our study was slightly lower (75.9%), the very high sensitivity supports its utility as a screening marker for neoplastic lesions. The high NPV of 95.7% for MUC1 expression in this study implies that a low MUC1 score (0/1) strongly indicates the absence of neoplasia, making it a valuable tool for ruling out malignancy in ambiguous cases. Conversely, its PPV of 75.9% supports its moderate ability to confirm neoplastic pathology when high expression is observed. These metrics collectively contribute to an overall diagnostic accuracy of 84.6% for MUC1, affirming its reliability in histopathological evaluation.

In contrast, MUC5AC demonstrated lower diagnostic performance. Although low expression (score 0/1) was significantly more frequent in neoplastic lesions (69.6%), its overall accuracy was only 71.2%, and both sensitivity and specificity hovered in the low 70% range. In line with our findings, Bhoge et al. reported that negative MUC5AC expression had 62.5% sensitivity and 93.3% specificity in distinguishing neoplastic lesions from calculous cholecystitis, and 62.5% sensitivity and 73.1% specificity from acalculous cholecystitis [7]. Similarly, Xiong et al. reported MUC5AC positivity in 94.3% of non-neoplastic and 51.9% of malignant gallbladder lesions, further supporting its inverse relationship with malignancy [10].

These collective findings point to a complementary diagnostic role for MUC1 and MUC5AC. MUC1 is best utilized to identify and confirm neoplastic potential, especially in suspected malignant lesions, whereas MUC5AC is more indicative of benign or inflammatory pathology and may aid in excluding malignancy when strongly expressed. The inverse expression patterns of these two mucins support their combined use as a panel, enhancing diagnostic confidence in histologically ambiguous cases.

Despite these findings, our study did not reveal significant associations between MUC1 or MUC5AC expression and histopathological features such as lymph node metastasis, LVI, PNI, TILs, or necrosis. This may be attributed to the limited number of GBC cases in our cohort. Bhoge et al. and other studies similarly failed to establish such correlations [7,26,27]. However, Xiong et al. and Hiraki et al. found significant associations of MUC1 expression with lymphatic invasion and lymph node metastasis, possibly due to their larger sample sizes (n = 108) [10,28].

Limitations and future perspectives

The primary limitation of this study is the relatively small sample size, particularly the limited number of GBC cases, which may affect the generalizability of the findings. Additionally, the absence of long-term follow-up data precludes assessment of the prognostic significance of MUC1 and MUC5AC expression. Another limitation is the lack of a control group with histologically normal gallbladder tissue, which could have clarified baseline MUC1 and MUC5AC expression and improved distinction between normal, reactive, and neoplastic mucosa. Future studies should involve larger, multicentric cohorts and incorporate survival analysis to validate these markers as prognostic tools. Molecular studies exploring the mechanistic pathways of MUC1 and MUC5AC in gallbladder carcinogenesis may further uncover their potential as therapeutic targets and improve personalized treatment strategies.

## Conclusions

This study underscores the diagnostic relevance of MUC1 and MUC5AC IHC expression in differentiating neoplastic from non-neoplastic gallbladder lesions. MUC1 was predominantly expressed in neoplastic and malignant conditions, highlighting its potential as a marker of malignancy and tumor aggressiveness. Conversely, MUC5AC was more frequently expressed in non-neoplastic lesions, suggesting its association with benign or early-stage pathology. The contrasting expression patterns of these mucins provide a useful tool for histopathological differentiation between benign and malignant gallbladder conditions. Incorporating MUC1 and MUC5AC into routine diagnostic panels may improve diagnostic accuracy and aid in clinical decision-making. Further large-scale, multicenter studies are recommended to validate their prognostic and therapeutic potential in gallbladder disease.
